# Common carotid arterial interadventitial distance (diameter) as an indicator of the damaging effects of age and atherosclerosis, a cross-sectional study of the Atherosclerosis Risk in Community Cohort Limited Access Data (ARICLAD), 1987–89

**DOI:** 10.1186/1476-7120-4-1

**Published:** 2006-01-03

**Authors:** Marsha L Eigenbrodt, Zoran Bursac, Kathryn M Rose, David J Couper, Richard E Tracy, Gregory W Evans, Frederick L Brancati, Jawahar L Mehta

**Affiliations:** 1 Department of Epidemiology, Fay W. Boozman College of Public Health, University of Arkansas for Medical Sciences, Little Rock, USA; 2 Department of Biostatistics, Fay W. Boozman College of Public Health, University of Arkansas for Medical Sciences, Little Rock, USA; 3 Department of Epidemiology, University of North Carolina at Chapel Hill, Chapel Hill, USA; 4 Department of Biostatistics, University of North Carolina at Chapel Hill, Chapel Hill, USA; 5 Department of Pathology, Louisiana State University Health Science Center, New Orleans, USA; 6 Department of Public Health Sciences, Wake Forest University School of Medicine, Winston-Salem, USA; 7 Departments of Medicine and Epidemiology, Johns Hopkins School of Medicine and Johns Hopkins Bloomberg School of Public Health, Baltimore, USA; 8 Departments of Internal Medicine, Physiology, and Biophysics, Director Division of Cardiovascular Medicine, University of Arkansas for Medical Sciences, Little Rock, USA

## Abstract

**Background:**

The effect of age on common carotid artery diameter is unclear for varying atherosclerosis risk levels.

**Methods:**

Cross-sectional data from the Atherosclerosis Risk in Communities Limited Access Data set were used to estimate the association of age with B-mode ultrasound common carotid artery diameter for three atherosclerosis risk levels. Based on information from clinical examinations, B-mode ultrasounds, questionnaires, blood and other tests, participants were categorized into three groups: pre-existing disease (prevalent stroke and/or coronary heart disease), high risk group (no pre-existing disease, but prevalent diabetes, hypertension, plaques/shadowing, body mass index >= 30, current smoking, or hyperlipidemia), and a low risk group (no pre-existing disease, no plaques/shadowing, and no major elevated risk factors). Multivariable linear regression analyses modeled the common carotid artery diameter relationship with age.

**Results:**

Age was positively and significantly associated with common carotid artery diameter after risk factor adjustment in the overall sample, but age had a larger effect among persons with evidence of atherosclerosis (interaction p < 0.05). Each year of older age was associated with 0.03 mm larger diameter/year among persons with pre-existing disease, with 0.027 mm larger diameter/year in the high risk group, but only 0.017 mm/year among the low risk group. Results were qualitatively similar using plaques/shadowing status to indicate atherosclerosis severity.

**Conclusion:**

The significant impact of age on common carotid artery diameter among low risk, middle-aged, black and white men and women suggests arterial remodelling may occur in the absence of identified risk factors. The significantly larger impact of age among persons with, compared to persons without identified atherosclerosis or its risk factors, suggests that arterial remodelling may be an indicator of exposure duration.

## Background

Because of its accessibility, the common carotid artery (CCA) is often evaluated using B-mode ultrasound to assess atherosclerosis severity [1-3]. Ultrasound, animal, and anatomic studies indicate arterial diameters are associated with hemodynamic factors [4-7] and with vascular damage [8,9]. Several studies have suggested that arterial wall area, or arterial diameter in conjunction with wall thickness, may provide useful information for understanding atherosclerosis progression, vascular injury, or vascular vulnerability [2,8,10-15]. However, the relationship between arterial wall thickness and diameter is not likely to be simple since both physiologic and pathological processes can contribute to diameter differences [13]. For example multiple factors such as gender [16], physiologic response to shear stress[17] as well as plaque characteristics [8,9] are known to be associated with arterial diameter. So, many factors could potentially impact the relationship between age and diameter. While many studies have evaluated associations of risk factors or vascular characteristics with arterial diameter [8,9,11,18-23], fewer studies have attempted to separate the effects of age and atherosclerosis on arterial diameter. The Bruneck study found that age was related to CCA diameter only in persons within the upper 50^th ^percentile of wall thickness [11] while a small study of 69 male subjects screened for the absence of atherosclerosis and its risk factors found that diameter did increase significantly with age [13].

This study extends previous studies of B-mode ultrasound CCA by estimating optimal B-mode ultrasound right CCA diameters (interadventitial distances) within a low-risk subset from a bi-racial population sample of both men and women; by determining whether atherosclerosis severity measures were effect modifiers of the age-CCA diameter relationship; and by estimating the effect of age among diseased, high risk, and low risk populations from the same population sample.

## Methods

### Study sample

For our analyses we used the ARICLAD set provided by the National Heart and Lung Institute (NHLBI) after the institutional review board at the University of Arkansas for Medical Sciences approved the secondary data analysis as having minimal risk to the participants within the Limited Access Data set. The ARIC study was previously approved by review boards at each institution involved in the original study. The ARICLAD is a subset of the ARIC Cohort Study, a population based study of atherosclerotic disease in 15,792 black and non-black men and women, ages 45 to 64, from 4 clinical centers: Jackson, Mississippi; Forsyth County, North Carolina; northwest Minneapolis suburbs; and Washington County, Maryland. Participants from the Jackson center were all black, about 14% of those from the Forsyth center were black, and the other participants were predominantly white (non-black) [24]. The sampling strategy for the ARIC Cohort Study has been reported previously [24]. As per NHLBI policy [25], the ARICLAD set excluded participants whose informed consents were not consistent with release to investigators beyond the original ARIC study, and after "records with personal identifiers and other variables that might enable individual participants to be identified, such as outliers, dates, and study sites, were removed or otherwise modified" leaving 15,732 (99.6%) baseline participants. From this set, a computer-generated random sample of 10,000 participants was chosen for a developmental data set, of which 9,109 participants had B-mode ultrasound diameter measurements of the right common carotid artery (CCA).

### B-mode ultrasound

At baseline, questionnaires were administered [24] and fasting blood samples were drawn and processed as previously described [26]. Trained and certified sonographers at each of the four ARIC clinical centers obtained B-mode ultrasounds of the right and left carotid artery from three carotid segments (CCA, bifurcation, and internal carotid artery) with the participant in a supine position [27,28]. Each scan was read at a central reading center to achieve maximal standardization of readings as previously described [28]. This study evaluated the diameters (inter-adventitial distances) obtained from the "optimal" angle of interrogation of the distal 1 cm segment of the CCA (i.e. the 1-cm segment proximal to the dilatation of the carotid bulb). The "optimal" angle of interrogation was defined as the longitudinal view that best displayed the Y-shaped origin of the internal and external carotids from the carotid bifurcation and the four interfaces needed to define near and far wall thicknesses [27]. Since an earlier study had shown coefficients describing the relationship between CCA diameter and risk factors to be essentially the same whether right, left, or both CCA diameters were used [29], we used the diameters from the right CCA because more data were available.

### Disease and subset definitions

Analyses were cross-sectional and evaluated either the cohort sample or cohort subsets that were categorized using baseline characteristics to represent populations with varying prevalence of atherosclerotic disease and its risk factors. The pre-existing disease subset was defined by prevalence of either coronary heart disease (CHD) or stroke at baseline. A high risk subset consisted of persons without prevalent CHD or stroke, but with atherosclerotic plaques/shadowing, diabetes, hypertension, obesity, current smoking, or hyperlipidemia. Prevalent CHD was based on electrocardiographic evidence of a myocardial infarction (MI), self-reported physician diagnosis of a MI, or history of a cardiac procedure at the baseline examination [30]. An algorithm based on a standardized, validated, stroke symptom questionnaire [31] and self-reported diagnosis of stroke at baseline was used to identify prevalent stroke. Plaques or calcification (shadowing) of the right or left carotid sites (internal, bifurcation, or common carotid arteries) were identified based on B-mode ultrasound wall shape, texture, and thickness as described previously [28]. Other major risk factors at baseline were identified as follows: prevalent diabetes (self-reported physician diagnosis, medication within 2 weeks, or fasting blood glucose ≥ 126 mg/dL, or non-fasting blood glucose ≥ 200 mg/dL); hypertension (anti-hypertensive medication within 2 weeks or BP ≥ 140/90); obesity (body mass index ≥ 30, computed as weight in kg divided by height in meters^2^); current cigarette smoking status; hyperlipidemia (LDL cholesterol ≥ 160 mg/dL and/or use of cholesterol lowering medication). Cigarette years of smoking were calculated as the product of the average number of cigarettes smoked per day times the number of years smoked. The low risk subset consisted of persons remaining after excluding the pre-existing disease and high risk subsets.

An alternative 2-level classification of atherosclerosis severity, based on the presence or absence of atherosclerotic plaques/shadowing, was evaluated in statistical models with and without the first severity variables (pre-existing disease and high risk status) with which there was overlap. These analyses were limited to persons with information on plaques or shadowing at any carotid site (n = 7,381).

### Statistical analysis

The analyses were performed using SAS v9.1.2 (SAS Institute Inc., Cary, N.C.). Baseline characteristics between disease severity groups were compared using one-way analysis of variance (ANOVA) and chi-square tests. Multiple linear regression analyses were used to determine both the parameter estimates for age and for atherosclerosis severity variables after adjustment for height, race, and gender (basic adjustment) and after adding statistically significant (p ≤ 0.05) atherosclerotic risk or preventive factors from the following list: body mass index (BMI), hypertension, diabetes, systolic and diastolic blood pressures (SBP and DBP), smoking status, drinking status, usual grams of alcohol consumption per week, LDL and HDL cholesterols, cholesterol lowering medication use, white blood count, and fibrinogen. To test for effect modification, variables representing interaction terms between age, gender, and race with variables for atherosclerosis severity were tested and retained at an alpha of 0.05. Adjustments were to the sample covariate means unless specified otherwise. Age centering was used in conjunction with interaction terms to estimate the effects of atherosclerosis severity at specific ages.

The gender-, height-, and age-specific means were estimated with ordinary least squared regression models adjusting for race (to the proportion in the low risk subset). The 5^th ^and 95^th ^percentiles around the means were estimated using quantile (percentile) regression available in experimental SAS procedure QUANTREG.

## Results

Of the 9,109 participants with right CCA diameter B-mode ultrasound measurements, 8,528 participants had adequate information for classification of disease severity and 8,163 participants had data for risk adjustment. Among persons with data from all 6 carotid sites (right or left, internal, bifurcation, and CCA), 36% were found to have plaque or shadowing (evidence of calcification) of at least 1 site. The low risk, high risk, and pre-existing disease groups were comprised of 1,397, 6175 and 591 participants respectively.

### Characteristics of study population and subsets

Characteristics used to define the pre-existing disease, high risk, and low risk subsets varied as expected (Table 1): smoking status, lipid profiles, and BP were most favorable in the low risk group, and heart disease and stroke were present only in the pre-existing group. However, other characteristics varied as well. The mean age, proportion of men, proportion of blacks, white blood count, and fibrinogen values were lowest in the low risk subset and were highest in the pre-existing disease subset except for black race. The low risk subset also had the largest proportion of current drinkers and the lowest ethanol consumption reported by drinkers. The group with pre-existing heart disease or stroke contained a greater proportion of former smokers, former drinkers, diabetics, hypertensive persons, users of cholesterol-lowering medication, and persons with plaques/shadowing, but a smaller proportion of current smokers, current drinkers, never smokers, and never drinkers than the high risk group. The pre-existing disease group also had a less favorable lipid profile (higher LDL and lower HDL), a lower DBP, and higher cigarette years smoked for current smokers than the high risk subset.

**Table 1:** Characteristics of the cohort sample and the cohort subsets*: pre-existing disease, high risk, and low risk subsets, the Atherosclerosis Risk in Communities Cohort Limited Access Data, 1987–89.

	Cohort Sample N = 8163^‡^	Subsets* of Cohort Sample			
		
		Pre-existing Disease N = 591^‡^	High Risk N = 6175^‡^	Low Risk N = 1397^‡^	p-value
Age (mean, std)	54.2 (5.7)	56.5 (5.3)	54.4 (5.7)	52.2 (5.4)	<0.0001
Male Gender (%)	44.8	64.3	44.8	36.7	<0.0001
Black Race (%)	25.5	25.5	28.7	12.3	<0.0001
Height in cm (mean, std)	168.5 (9.3)	170.4 (8.9)	168.4 (9.3)	168.0 (9.0)	<0.0001
Stroke (%)	2.4	33.7^†^	0^§^	0^§^	NA
Coronary Heart Disease	5.2	72.2^†^	0^§^	0^§^	NA
Plaque or Shadowing	35.7	52.7	44.0^†^	0^§^	0.0002
Smoker (%)					
Current	26.9	29.6	32.8^†^	0^§^	0.0783^§§^
Former	31.8	43.8	28.7	40.5	
Never	41.3	26.6	38.5	59.5	
Cigarette Years for Current Smokers (mean, std)	681.9 (438.9)	845.3 (532.4)	667.7 (427.1)	N/A	<0.0001
Drinker (%)					
Current	56.3	48.6	55.4	63.4	<0.0001^§§^
Former	19.3	31.5	19.1	14.9	
Never	24.4	20.0	25.5	21.7	
Ethanol g/wk for Current Drinkers (mean, std)	76.3 (119.1)	87.8 (125.5)	81.1 (125.8)	54.1 (81.9)	<0.0001
Diabetes (%)	11.4	22.3	12.9^†^	0^§^	<0.0001
Hypertension (%)	35.5	54.3	41.7^†^	0^§^	<0.0001
Cholesterol Medication Use (%)	3.0	6.5	3.3^†^	0^§^	<0.0001
Body Mass Index (mean, std)	27.7 (5.3)	28.2 (5.4)	28.3 (5.5)^†^	24.7 (2.8)	<0.0001
Systolic Blood Pressure mm Hg (mean, std)	121.4 (18.7)	122.7 (21.6)	123.5 (19.0)^†^	111.5 (12.0)	<0.0001
Diastolic Blood Pressure mm Hg (mean, std)	73.7 (11.3)	72.6 (12.5)	74.8 (11.5)^†^	69.6 (8.5)	<0.0001
LDL Cholesterol mg/dl (mean, std)	138.2 (39.5)	145.2 (39.5)	142.5 (40.3)^†^	116.2 (26.0)	<0.0001
HDL Cholesterol mg/dl (mean, std)	51.5 (17.0)	44.6 (15.9)	50.4 (16.2)	59.2 (18.7)	<0.0001
Fibrinogen (mean, std)	304.3 (65.3)	321.9 (72.0)	308.6 (65.0)	277.5 (55.5)	<0.0001
White Blood Count (thousands) (mean, std)	6.2 (2.1)	6.7 (3.3)	6.3 (2.0)	5.4 (1.5)	<0.0001

*Subsets: Pre-existing disease = prevalent CHD or stroke; High risk = absence of pre-existing disease + either diabetes, hypertension, body mass index ≥ 30, current cigarette smoking, LDL cholesterol ≥ 160 mg/dL, use of cholesterol lowering medication, or presence of plaque or shadowing on B-mode ultrasound; Low risk = absence of pre-existing disease and risk factors.

Percentages may not total to 100% because of rounding.

^‡^N = maximum possible in the cohort or subsets. For some variables, smaller numbers are available because of missing values.

^†^Variable levels were used to distinguish between the high risk factor and low risk subsets.

N/A since smokers were excluded from low risk subset.

^§^Subset was defined by the absence of the characteristic.

^§§^Comparison of equality of proportions of current smokers and current drinkers between groups.

### Age-diameter relationships: adjustment to study population means

When disease severity dummy variables (pre-existing disease, high risk status) were used as indicators of atherosclerosis severity, the risk-adjusted models retained age, race, height, BMI, current smoker status and cigarette years of smoking, current drinker status and usual ethanol intake, SBP, and DBP, and fibrinogen. When interactions were tested, the age-diameter relationships were not the same among the pre-existing disease, high risk and low risk subsets (p < 0.05 for deviance tests comparing models with and without interaction terms). Therefore, interaction terms (age-high risk status and age-pre-existing disease status) were retained and independent variable betas are shown in Table 2.

**Table 2:** Beta coefficients and 95% confidence intervals (CI) for multivariable linear regression models with CCA diameter as the outcome after adjustment for basic covariates and after adding atherosclerotic risk factors.* Both models included interaction terms allowing determination of effects of age in subsets of varying atherosclerosis severity^†^, the Atherosclerosis Risk in Communities (ARIC) Limited Data Set, 1987–1989

	Basic Model N = 8163		Full Model N = 8163	
	Beta (95% CI)	p-value	Beta (95% CI)	p-value

Main effects				
Male gender	0.492 (0.439, 0.545)^‡^	<0.0001	0.435 (0.384, 0.486)^‡^	<0.0001
Black race/ethnicity	0.190 (0.149, 0.231)^‡^	<0.0001	0.087 (0.044, 0.130)^‡^	<0.0001
Height (cm)	0.020 (0.018, 0.022)^‡^	<0.0001	0.023 (0.021, 0.025)^‡^	<0.0001
Current smoking	-		0.154 (0.107, 0.201)^‡^	<0.0001
Current drinking	-		-0.038 (-0.075, -0.001)^‡^	0.0485
Cigarette years of smoking (in 100's)	-		0.019 (0.015, 0.023)^‡^	<0.0001
Ethanol (100 grams/week)	-		0.039 (0.021, 0.057)^‡^	<0.0001
BMI	-		0.025 (0.021, 0.029)^‡^	<0.0001
Systolic BP	-		0.017 (0.015, 0.019)^‡^	<0.0001
Diastolic BP	-		-0.013 (-0.015, -0.011)^‡^	<0.0001
Fibrinogen	-		0.0002 (0.0000, 0,0004)^‡^	0.0335
Age (1 year of age)	0.028 (0.020, 0.036)^†^	<0.0001	0.017 (0.009, 0.025)^†^	<0.0001
Additional diameter increase per year of age				
Among Diseased Subset	0.018 (0.004, 0.032)^§^	0.0139	0.013 (-0.001, 0.027)^§^	0.0611
Among Risk Factor Subset	0.011 (0.003, 0.019)^§§^	0.0096	0.011 (0.003, 0.019)^§§^	0.0101

* Basic model independent variables = age, race, gender, height, dummy variables for disease severity, and interaction terms between age-disease severity dummy variables. Full model added variables = BMI, systolic and diastolic blood pressures, current smoker status, cigarette years of smoking in hundreds, current drinker status and grams of ethanol usually consumed per week in 100 g, fibrinogen value.

^‡^Beta indicates the expected difference in millimeters of CCA diameter associated with the presence of each categorical variable and for each unit increase of continuous variables.

^†^Beta indicates the expected difference in millimeters of CCA diameter for each year of older age in the low risk subset.

^§-§§^Betasindicate the expected difference in millimeters of CCA diameter associated with each year of age among the diseased (^§^) and risk factor or high risk (^§§^) subsets.

In basic models (Table 2), men (beta = 0.492) and blacks (beta = 0.19) had significantly larger CCA diameters than women and whites respectively. Height (beta = 0.02) was significantly and positively associated with CCA diameter as well. Adjusting for atherosclerotic risk factors (Table 2, full model) resulted in a reduction of the effect sizes associated with race (beta = 0.087) and gender (beta = 0.435) by approximately 54% and 12% respectively, but did not reduce the effect of height (beta = 0.023).

In the basic model, each year of older age was associated with almost 0.03 mm larger CCA diameter (Table 2, age main effect) in the low risk subset, but the age effect was larger among the diseased (main effect + interaction term = 0.046 mm/year) and high risk subsets (main effect + interaction term = 0.039 mm/year) (Table 2). Not shown in the table is that by age 64, CCA diameters were 0.602 mm (SE = 0.077) larger in the pre-existing disease and 0.427 mm (SE = 0.056) larger in the high risk subsets than in the low risk subset prior to risk adjustment (both p < 0.0001). After risk factor adjustment, the betas were markedly reduced but still significant (beta = 0.252 and beta = 0.14 for the pre-existing disease and high risk group respectively, both p < 0.01). Except for current drinking status and DBP, all covariates were positively associated with CCA diameter (Table 2).

### Age-diameter relationships: separate models adjusting to young-age risk levels

**Figure 1:**
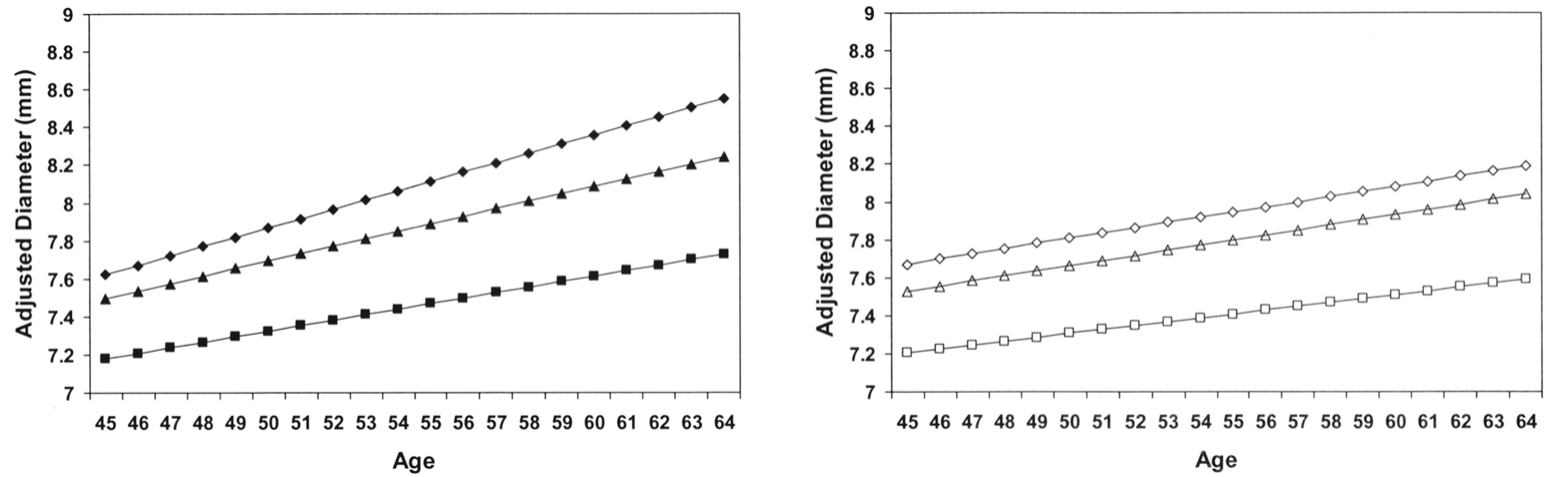
Age-specific, adjusted B-mode ultrasound common carotid artery diameters for study subsets, ARIC Limited Access data, 1987–89. *Age-specific diameters were estimated for each risk subset using betas determined from stratified models after adjusting for basic variables (race, sex, and height) and after adding risk factors (body mass index, SBP, DBP, current smoker status, usual ethanol intake (gms/week), fibrinogen, and years of smoking). **Adjustment was to the covariate means/proportions of persons age 45 to 50 of each subset. Diamonds indicate pre-existing disease subset; triangles indicate high risk subset; squares indicate low risk subset; Solid symbols indicate basic adjustment and open symbols indicate risk factor adjustment.

Results were similar when the relationship between age and diameter was determined for each subset in separate analyses (Figure 1). Betas for covariates were determined in linear regression models for each subset. Each stratified analysis was then adjusted to the means/proportions for the covariates of persons who were 45 to 50 years old in the respective subsets. Older age was associated with larger diameters in all subsets regardless of the level of adjustment, but the effect of age was reduced by risk factor adjustment in all subsets (slopes decreased after adjustment). Diameters for the pre-existing disease and high risk groups were larger than the low risk group, regardless of the level of adjustment. After risk factor adjustment, there was little difference between the slopes for the pre-existing disease and high risk groups (pre-existing disease: 0.028 mm/year; high risk group: 0.027 mm/year), but both groups continued to have slightly greater slopes than the low risk group (0.020 mm/year).

### Age-diameter relationships: plaque/shadowing as indicator of atherosclerosis severity

The alternative measure of atherosclerosis severity (plaque/shadowing presence or absence) was also found to be an effect modifier of the age-CCA diameter relationship in both basic (p = 0.01) and risk factor adjusted models (p = 0.02). After basic adjustment, age was positively associated with CCA diameter (0.035 mm/year) when plaques were absent (p < 0.0001) and with an additional 0.009 mm/year when plaques were present (p = 0.0145). Risk factor adjustment reduced the effect of age for persons without plaques (beta = 0.021, SE = 0.002, p < 0.0001) and reduced the added age effect found when plaques were present (beta = 0.008, SE = 0.003, p = 0.02).

### Models with plaque/shadowing and disease/risk status

In statistical models with basic adjustment but no interaction terms, plaque/shadowing status, and pre-existing disease and high risk status were significantly associated with CCA diameter (p < 0.0001). The addition of risk factors (current smoking status and cigarette years of smoking, drinking status and ethanol consumption, body mass index, fibrinogen, and SBP and DBP) resulted in a loss of significance of pre-existing disease (beta = 0.022, SE = 0.044, p = 0.6) and high risk status (beta = -0.039, SE = 0.028, p = 0.16), but not in plaque status (beta = 0.148, SE = 0.021, p < 0.0001). After adjustment, plaque status remained a significant modifier of the age effect on diameter (p < 0.03).

### Estimates of age-, gender-, and height-specific right CCA diameter

Age had the smallest impact on the right CCA diameter among the low risk subset. Table 3 provides the gender-, height-, and age-specific mean diameters and corresponding percentiles for the low risk subset after adjusting for race. Right CCA diameters were larger for men, for tall persons, and for older persons compared to their respective counterparts.

**Table 3:** The age-specific means and 5^th ^and 95^th ^percentiles for men and women by height categories, adjusted for race* determined from low risk participants of the ARIC Limited Access Data set, 1987–89.

***Men***									
	*Height <170 cm*			*Height 170–179 cm*			*Height 180+ cm*		

*Age*	*Mean Diameter*	*Diameter Percentiles*		*Mean Diameter*	*Diameter Percentiles*		*Mean Diameter*	*Diameter Percentiles*	
		*5*^*th*^	*95th*		*5*^*th*^	*95th*		*5*^*th*^	*95th*

45	7.45	6.60	8.15	7.59	6.74	8.85	7.84	6.91	8.90
46	7.48	6.62	8.19	7.62	6.75	8.90	7.86	6.90	8.92
47	7.51	6.65	8.23	7.65	6.76	8.94	7.88	6.88	8.95
48	7.53	6.68	8.28	7.69	6.78	8.99	7.90	6.86	8.98
49	7.56	6.70	8.32	7.72	6.79	9.04	7.92	6.85	9.00
50	7.59	6.73	8.36	7.75	6.80	9.08	7.95	6.83	9.03
51	7.61	6.76	8.40	7.78	6.81	9.13	7.97	6.81	9.05
52	7.64	6.78	8.45	7.81	6.83	9.18	7.99	6.80	9.08
53	7.67	6.81	8.49	7.84	6.84	9.22	8.01	6.78	9.11
54	7.69	6.83	8.53	7.87	6.85	9.27	8.03	6.76	9.13
55	7.72	6.86	8.58	7.91	6.86	9.32	8.05	6.75	9.16
56	7.75	6.89	8.62	7.94	6.88	9.36	8.07	6.73	9.19
57	7.77	6.91	8.66	7.97	6.89	9.41	8.09	6.71	9.21
58	7.80	6.94	8.70	8.00	6.90	9.46	8.11	6.70	9.24
59	7.83	6.97	8.75	8.03	6.91	9.50	8.13	6.68	9.26
60	7.85	6.99	8.79	8.06	6.93	9.55	8.15	6.66	9.29
61	7.88	7.02	8.83	8.09	6.94	9.60	8.17	6.65	9.32
62	7.91	7.05	8.88	8.13	6.95	9.64	8.19	6.63	9.34
63	7.93	7.07	8.92	8.16	6.96	9.69	8.21	6.62	9.37
64	7.96	7.10	8.96	8.19	6.98	9.74	8.23	6.60	9.39

***Women***									

	*Height <160 cm*			*Height 160–169 cm*			*Height 170+ cm*		

*Age*	*Mean Diameter*	*Diameter Percentiles*		*Mean Diameter*	*Diameter Percentiles*		*Mean Diameter*	*Diameter Percentiles*	
		*5*^*th*^	*95th*		*5*^*th*^	*95th*		*5*^*th*^	*95th*

45	6.82	6.13	7.75	6.93	6.09	7.93	7.15	6.17	8.10
46	6.84	6.13	7.77	6.97	6.12	7.96	7.18	6.20	8.15
47	6.86	6.13	7.79	7.0	6.15	7.99	7.21	6.22	8.21
48	6.88	6.14	7.81	7.03	6.18	8.02	7.25	6.25	8.26
49	6.90	6.14	7.83	7.06	6.21	8.06	7.28	6.28	8.31
50	6.93	6.14	7.85	7.10	6.24	8.09	7.31	6.30	8.37
51	6.95	6.15	7.87	7.13	6.27	8.12	7.35	6.33	8.42
52	6.97	6.15	7.89	7.16	6.29	8.15	7.38	6.36	8.47
53	6.99	6.15	7.91	7.20	6.32	8.18	7.41	6.39	8.53
54	7.01	6.16	7.93	7.23	6.35	8.22	7.45	6.41	8.58
55	7.03	6.16	7.94	7.26	6.38	8.25	7.48	6.44	8.63
56	7.05	6.16	7.96	7.30	6.41	8.28	7.51	6.47	8.69
57	7.08	6.17	7.98	7.33	6.44	8.31	7.55	6.49	8.74
58	7.10	6.17	8.00	7.36	6.47	8.35	7.58	6.52	8.79
59	7.12	6.17	8.02	7.39	6.50	8.38	7.61	6.55	8.85
60	7.14	6.18	8.04	7.43	6.53	8.41	7.64	6.57	8.90
61	7.16	6.18	8.06	7.46	6.56	8.44	7.68	6.60	8.95
62	7.18	6.18	8.08	7.49	6.59	8.48	7.71	6.63	9.00
63	7.20	6.19	8.10	7.53	6.62	8.51	7.74	6.66	9.06
64	7.23	6.19	8.12	7.56	6.65	8.54	7.78	6.68	9.11

*Diameters adjusted to the same proportion of black participants (13%)

## Discussion

This study provides estimates for the effect of age on the right CCA diameter in both low and high risk populations. As an estimate of optimal right CCA diameters for middle-aged adults, the study provides age-, gender- and height-specific estimates for right CCA diameters from a low risk subset of the ARICLAD. Our findings of a significant, positive association between age and CCA diameter among a low risk population, with a larger effect among persons with atherosclerotic disease or high levels of risk factors, suggest that arterial remodeling may indicate duration of exposure among persons with atherosclerosis or elevated risk factors and duration of "normal" stresses among persons with low risk factor levels.

That atherosclerosis and atherosclerotic risk factors are associated with arterial diameter is well recognized [7,20,29,32], and recent studies have suggested arterial remodelling frequently occurs in association with vulnerable plaques [8,9,14]. So, associations between age and arterial diameter may not reflect the contribution of age itself, but instead, an increase in prevalence and/or severity of atherosclerosis or its risk factors with increasing age. The prospective Bruneck study, comprised of a random sample of men and women 40 to 85 years old, found a statistically significant increase in CCA diameter with age for persons in the highest 50^th ^percentile of wall thickness, but a much smaller and non-significant diameter increase among persons with thinner CCA walls [11]. Similarly, our study found a larger effect of age on CCA diameter in persons with pre-existing atherosclerotic disease or high cardiovascular risk factor levels compared to low risk persons (e.g. without atherosclerotic disease and with low CVD risk factor levels). In our study, the greater effect of age on the CCA diameter in the higher risk groups was driven by the presence of atherosclerotic plaques. In contrast to the Bruneck study, our study found a significant positive association between age and CCA diameter even in the low risk subset. A number of differences between the studies could explain the discrepant findings: our study evaluated cross-sectional data and the Bruneck study evaluated prospective data; our study was of the right CCA and the Bruneck study combined right and left CCA; different criteria were used to define the low risk groups; and the study populations were different. However, qualitatively, results from the two studies are similar. CCA did increase with age in the Bruneck study low risk group, just not significantly so. Another study evaluated 69 male patients and athletes, aged 16–75, who were selected for the absence of major atherosclerotic risk factors and plaque [13]. In this convenience sample, age was a strong independent predictor of CCA diastolic diameter. Each ten years of increasing age was associated with a 0.17 mm larger CCA diameter [13], which is similar to the estimate in persons without atherosclerotic plaques (0.02 mm/year) and identical to the risk factor adjusted estimate from our low risk subset (0.017 mm/year). These findings support an effect for aging that is separate from that of clinically identifiable atherosclerosis and which appears to be relatively uniform in middle aged populations without major risk factors.

The significant interactions between both atherosclerosis severity measures and age in models predicting CCA diameter are consistent with age being a marker for length of exposure to the damaging risk factor effects. However, a significant interaction persisted only between age and plaques after adjustment for risk factors suggesting that much of the greater age effect in the pre-existing disease and high risk groups occur because of the effect of atherosclerotic damage evidenced by the presence of plaques.

The low risk subset was selected so that subjects had risk factor values lower than those typically used to identify cardiovascular risk. The reduction in the parameter estimate for age's impact on CCA diameter in the low risk group after adjusting for continuous atherosclerotic risk factors, indicates that even risk factor levels not commonly classified as detrimental can impact CCA diameters. This is not surprising given previous findings for BP [33]. Thus, at least part of the CCA diameter differences seen with older age in this low risk group was likely due to higher (though still normal) atherosclerosis risk factor levels at older ages. The persistence of a significant, positive association between age and CCA diameter in the low risk population, even after adjustment for atherosclerotic risk factors, suggests that damage or weakening of the arterial wall associated with aging also contributes to diameter enlargement. Since the primary goal of the ARIC ultrasound examination was not to identify plaques, we cannot exclude the possibility that some of the CCA diameter enlargement, even in the low risk group, was due to underlying atherosclerosis which was not identified.

This difference in diameters among the low risk group and higher risk groups has important implications for defining normal values in general. Since the prevalence of obesity and associated risk factors is high in many populations [34-36], using population based samples could result in incorrect estimations of optimal values for CCA diameter and other risk factors influenced by obesity. Thus, persons at risk could be misclassified as being normal, and the possibility of misclassification could be largest in populations with the greatest obesity and risk.

Because the study is cross-sectional in design, the associations that were identified do not necessarily indicate a causal mechanism. The division into low risk, high risk and pre-existing disease groups is somewhat arbitrary; and so, results could reflect differences because of the ages represented within the risk groups. However, given that substantial proportions of all age categories 45–49, 50–54, 55–59, and 60–64 were represented within each risk group, age differences are unlikely to be driving the findings. Persons who had missing information leading to their exclusion in the current study were different in a number of ways from the persons available for study. However, since the associations for age and CCA diameter were quantitatively similar to those reported by for a smaller "normal" male population and qualitatively similar to those reported in a prospective study [11,13], our results are likely to provide reasonable estimates for the differences that would be found in populations with disparate atherosclerotic risk.

## Conclusion

In conclusion, consistent with our hypothesis and previous studies, our cross-sectional results indicate that persons with pre-existing atherosclerotic disease or atherosclerosis risk factors have larger diameters than persons without those attributes. More importantly, we also found that the impact of each year of older age on CCA diameter was significantly larger among populations with pre-existing cardiovascular disease and among persons with high risk factor levels or plaques compared to those at low risk of atherosclerotic disease and we were able to quantify the expected effect. Future investigations should consider the relationship between age, length of risk factor exposure, diameter, wall thickness, plaque, and disease development to better understand the progression of atherosclerosis and development of cardiovascular disease.

## List of abbreviations

CCA: common carotid artery

ARICLAD: Atherosclerosis Risk in Communities Limited Access Data

CHD: coronary heart disease

MI: myocardial infarction

LDL: low density lipoprotein

HDL: high density lipoprotein

SBP: systolic blood pressure

DBP: diastolic blood pressure

NHLBI: National Heart, Lung, and Blood Institute

## Authors' contributions

MLE developed the study proposal, performed some analyses and drafted and revised the manuscript.

ZB provided most analyses and provided input into manuscript revisions.

DJC and GE provided input into the analyses while GE also provided understanding of the ARIC B-mode ultrasound procedures.

RET, FB, KR, and JLM provided knowledge into the pathogenesis of atherosclerosis and revisions of the manuscripts.

## Disclosures

The authors have no conflicts of interest to disclose. Neither the interpretation nor presentation of the data was influenced by personal or financial relationship with other people or organizations.
